# Managing uncertainty in movement knowledge for environmental decisions

**DOI:** 10.1111/conl.12620

**Published:** 2018-12-03

**Authors:** Annabel L. Smith, Heini Kujala, José J. Lahoz‐Monfort, Lydia K. Guja, Emma L. Burns, Ran Nathan, Erika Alacs, Philip S. Barton, Sana Bau, Don A. Driscoll, Pia E. Lentini, Alessio Mortelliti, Ross Rowe, Yvonne M. Buckley

**Affiliations:** ^1^ School of Natural Sciences, Zoology, Trinity College Dublin The University of Dublin Dublin 2 Ireland; ^2^ School of Biosciences The University of Melbourne Melbourne Australia; ^3^ Parks Australia Division, Department of the Environment and Energy Australian Government Canberra Australia; ^4^ Centre for Australian National Biodiversity Research CSIRO Canberra Australia; ^5^ Fenner School of Environment and Society Australian National University Canberra Australia; ^6^ Long Term Ecological Research Network Terrestrial Ecosystem Research Network Canberra Australia; ^7^ Department of Ecology, Evolution and Behavior, The Alexander Silberman Institute of Life Sciences The Hebrew University of Jerusalem Jerusalem Israel; ^8^ Wildlife Heritage & Marine Division, Department of the Environment and Energy Australian Government Canberra Australia; ^9^ School of Life and Environmental Sciences Deakin University Geelong Burwood Victoria Australia; ^10^ Department of Wildlife, Fisheries, and Conservation Biology University of Maine Orono Maine USA; ^11^ Environment Standards Division, Department of the Environment and Energy Australian Government Canberra Australia; ^12^ School of Biological Sciences University of Queensland St Lucia Queensland Australia

**Keywords:** biodiversity conservation, connectivity, corridors, decision theory, dispersal, environmental policy, movement ecology, science‐policy interface

## Abstract

Species’ movements affect their response to environmental change but movement knowledge is often highly uncertain. We now have well‐established methods to integrate movement knowledge into conservation practice but still lack a framework to deal with uncertainty in movement knowledge for environmental decisions. We provide a framework that distinguishes two dimensions of species’ movement that are heavily influenced by uncertainty: *knowledge* about movement and *relevance* of movement to environmental decisions. Management decisions can be informed by their position in this knowledge‐relevance space. We then outline a framework to support decisions around (1) increasing understanding of the relevance of movement knowledge, (2) increasing robustness of decisions to uncertainties and (3) improving knowledge on species’ movement. Our decision‐support framework provides guidance for managing movement‐related uncertainty in systematic conservation planning, agri‐environment schemes, habitat restoration and international biodiversity policy. It caters to different resource levels (time and funding) so that species’ movement knowledge can be more effectively integrated into environmental decisions.

## 1 INTRODUCTION

Movement of organisms profoundly influences population and community dynamics (Nathan et al., 2008) and plays a critical role in how species and ecosystems respond to land‐use and climate change (Schloss, Nuñez, & Lawler, 2012). In recent years, there has been a flurry of progress in integrating plant and animal movement data into conservation science. We now have clear guidelines on linking movement attributes to management actions (Allen & Singh, 2016; McGowan et al., 2017), linking movement types (home range movement, dispersal, nomadic movement, and migration) to relevant policy domains (Barton et al., 2015) and using value of movement information in structured decision‐making (McGowan & Possingham, 2016).

Despite these developments, environmental decisions are still being made with limited movement knowledge because information on target organisms is unavailable, inaccessible or uncertain. Movement potential is often inferred indirectly from vegetation cover, occupancy, genetic structure or traits because directly studying movement is, or is perceived as being, expensive, time‐consuming, and difficult (Driscoll et al., 2014; Nguyen, Young, & Cooke, 2017). Political, cultural, and economic priorities often orient decisions, so gaining new movement knowledge can be given low priority (Schwartz et al., 2018).

A range of tools exist to aid environmental decision‐making, but decisions concerning species’ movement require a specific way of thinking. Confusion exists among practitioners about the distinction between uncertainty in movement parameters and the relevance of movement to different decisions. This is because different movement parameters (e.g., home range size vs. dispersal kernel) relate to management questions at vastly different scales (Barton et al., 2015). The confusion is compounded by ongoing debate about appropriate functional forms for movement parameters (Cousens, Hughes, & Mesgaran, 2018). Some decisions, such as reserve design, might require quantitative robustness analysis of severe uncertainty (Moilanen et al., 2006) while others, such as invasive species management, require value‐of‐information and sensitivity analyses before reliable decisions can be made (Moore & Runge, 2012). Decisions involving species’ movement however, might often need to consider multiple strategies for managing uncertainty. Ignoring uncertainty about movement could lead to inappropriate decisions with wasted resources and potentially perverse outcomes for biodiversity (Halpern, Regan, Possingham, & McCarthy, 2006). There is thus a critical need for guidance in making environmental decisions and developing policy when movement knowledge is uncertain.

In this article, we provide a framework that distinguishes two dimensions of species’ movement that are heavily influenced by uncertainty: (1) *knowledge* about the movement of propagules, genes, and individuals and (2) the *relevance* of these movements to environmental decisions. We draw on existing taxonomies of uncertainty (Higgins et al., 2003; Kujala, Burgman, & Moilanen, 2013; Regan, Colyvan, & Burgman, 2002) to define a knowledge‐relevance space which clarifies the state of movement knowledge as a first step to decision‐making. We then present a framework to manage uncertainty in movement knowledge for environmental decisions and illustrate the framework using a real‐world case study.

## 2 IDENTIFYING MOVEMENT‐RELATED UNCERTAINTY

**Figure 1:**
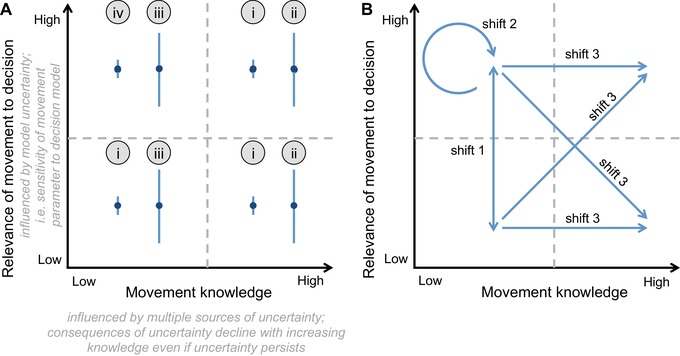
A framework for identifying a position in the knowledge‐relevance space as a starting point for decision‐making. (A) There are two dimensions of species’ movement that are influenced by different types of uncertainty, represented by two continuous axes: movement knowledge and relevance of movement to decisions. Error bars along the *y*‐axis describe how relevance can be influenced by the sensitivity of a movement parameter to a decision. We defined eight potential uncertainty scenarios (blue points) which relate to four “strategy options” (circled numbers, described in white boxes on Figure 2). The strategy options link Figures 1 and 2: the initial position on Figure 1A determines actions to reduce uncertainty (Figure 2), which can shift the position in knowledge‐relevance space (Figure 1B). (B) There are three ways decision‐makers can shift within the knowledge‐relevance space. Shift 1: clarify the relevance of movement knowledge (sensitivity analysis); shift 2: increase robustness of decisions to uncertainties (without reducing uncertainty); and shift 3: improve movement knowledge

We define two dimensions of species’ movement that are heavily influenced by uncertainty, represented by two continuous axes (Figure 1). The *x*‐axis describes current knowledge of movement for a species or community targeted by management. Movement knowledge is influenced by several sources of epistemic uncertainty including measurement or systematic error, natural variation, or subjective interpretation of information (Kujala et al., 2013; Regan et al., 2002). In some cases, uncertainty in movement knowledge is severe and can only be parameterized by a best guess (Halpern et al., 2006). Linguistic and human‐derived uncertainty can also influence movement knowledge, including vague or context‐specific terms, subjective judgment, and personal beliefs (Kujala et al., 2013). Movement knowledge could be low even when information is abundant, for example if data are contradictory. Good movement knowledge implies that uncertainties in information and the underlying data are understood well enough to assist decision‐making. The consequences of uncertainty should therefore decline as movement knowledge increases, even if uncertainty persists (Figure 1).

The *y*‐axis (Figure 1) represents the relevance of a movement parameter to a decision and is influenced primarily by model uncertainty (a type of epistemic uncertainty in the representation of a biological process, Higgins et al., 2003; Kujala et al., 2013). We separated relevance from movement knowledge because it relates to the scale of movement and type of management decision (Barton et al., 2015) and it involves specific processes for reducing uncertainty (represented by the error bars on the *y*‐axis). Model uncertainty includes structural components (i.e., the type of model used to describe movement) and parametric components (the movement parameter, such as a dispersal distance and habitat configuration) (Kujala et al., 2013). The model could be quantitative, such as meta‐population or diffusion model, or a conceptual way of describing the relevant components in a system. Parametric uncertainty could be low, while model uncertainty remains high because we do not know how the parameter affects the decision. Movement knowledge is likely to be highly relevant for an invasive species management plan (e.g., Coutts, Yokomizo, & Buckley, 2013) but might be less relevant for increasing the reproductive success of an endangered species at a particular site (e.g., Runge, Converse, & Lyons, 2011). However, when model uncertainty exists, the relevance of movement knowledge to the decision is uncertain.

## 3 MANAGING MOVEMENT‐RELATED UNCERTAINTY

**Figure 2:**
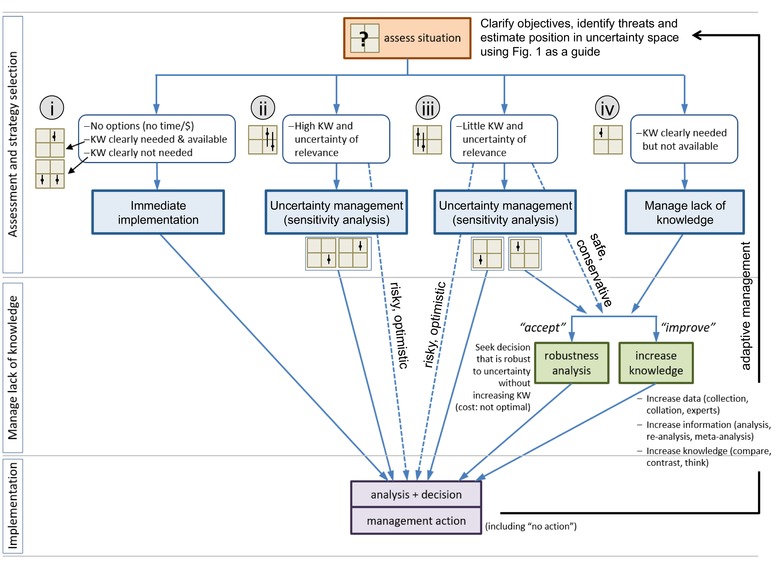
A decision‐support tool to help manage uncertainty in movement knowledge (KW) when making environmental decisions. Environmental decisions typically begin with objectives (e.g., to increase habitat connectivity), followed by identifying threats (e.g., isolation) that could be mitigated. Once these have been articulated, Figure 1 can be used to guide thinking about uncertainty in movement knowledge and its relevance to the decision. This places the decision‐maker at one of four “strategy options” (circled numbers), from which management pathways can be identified (blue arrows). The process then involves managing uncertainty in relevance, managing lack of knowledge and implementation

Our decision‐support framework (Figure 2) can assist environmental management decisions depending on movement knowledge and its relevance for decision‐making. The procedure is divided into three stages: (1) assessment of initial status and strategy selection (including uncertainty management), (2) managing lack of knowledge, if required, and (3) implementation (potentially including analysis, decision‐making and adaptive management). There are four “strategy options” that depend on levels of uncertainty in movement knowledge and relevance (Figure 1A). The first stage includes identifying opportunities to shift one's position in the knowledge‐relevance space (Figure 1B) and these shifts will influence the management pathway. Management can be immediately implemented in special cases when movement is clearly relevant to the decision and adequate movement knowledge is available, or, when movement is clearly irrelevant to the decision (strategy option i, Figures 1A and 2). When the relevance of movement knowledge is uncertain (strategy options ii and iii), management can also proceed immediately (dotted lines on Figure 2), but this carries a risk of either wasting resources (if movement is not relevant) or of ignoring movement and making a poor decision (if movement is relevant). Thus, safer options are to manage uncertainty or manage lack of knowledge. When movement knowledge is needed but not available (strategy option iv), the lack of knowledge must be managed.

### 3.1 Assessment and strategy selection

Figure 1A can help decision‐makers think critically about their initial position in the knowledge‐relevance space and to decide whether to: (1) increase understanding of the *relevance* of movement knowledge, (2) increase the *robustness* of decisions to uncertainties, or (3) increase knowledge of movement. Improved understanding of relevance will help decision‐makers reduce uncertainty on the *y*‐axis (the length of the vertical lines). As uncertainty declines, it might also change the position along the *x*‐axis. A change in position along the *x*‐axis occurs when movement knowledge increases, which might simultaneously improve understanding of the relevance of movement knowledge (diagonal arrows on Figure 1B).

Sensitivity analysis can clarify the relevance of movement to environmental decisions (strategy options ii and iii, Figure 2) which, in turn, might affect whether implementation can proceed directly or whether lack of knowledge should be managed (strategy option iii). In sensitivity analysis, different scenarios are investigated using values that span the possible range of, and sensitivity to, a given management option (shift 1, Figure 1B). Sensitivity analysis has shown that variation in seed dispersal kernels predicted movement rates of an invasive pine (*Pinus nigra*), leading to recommendations to prioritize spread prevention rather than controlling patch‐level demographic factors (Caplat, Nathan, & Buckley, 2012). It has also shown that climate change and disturbance regime influenced extinction risk of an endangered shrub (*Ceanothus verrucosus*) more than variation in movement (Lawson, Regan, Zedler, & Franklin, 2010).

### 3.2 Managing lack of knowledge

Value‐of‐information analysis can be used to direct resources most efficiently towards either gaining new information (*improve*) or implementing management based on available knowledge (*accept*) (Figure 2) (Canessa et al., 2015; McGowan et al., 2017). The outcome will be strongly influenced by funding and time. For example, value‐of‐information analysis showed that improving biological knowledge for koala conservation would only be cost‐effective with extremely large budgets that allowed expensive management decisions to be made (habitat restoration) over long time frames (Maxwell et al., 2015).

When very restricted time frames prevent even basic data collection and analysis, accepting uncertainty in movement knowledge might be necessary. In cases where movement knowledge is clearly relevant but movement knowledge is low (strategy option iv, Figure 2), robustness analysis can identify solutions that satisfy management goals, without resolving uncertainty (shift 2, Figure 1B). When uncertainty in a movement parameter is severe, info‐gap analysis can help in making robust environmental decisions by modeling a best estimate of movement and an unbounded uncertainty parameter around that estimate (Moilanen et al., 2006). For example, Halpern et al. (2006) quantified optimal spacing for marine reserves even though larval dispersal estimates for most marine species affected by the decision were severely uncertain. Increasing robustness can help identify a suitable strategy but it does not change the initial position in the knowledge‐relevance space because we do not gain new knowledge (shift 2, Figure 1B). Finding a more robust solution is nevertheless worthwhile as it requires that uncertainty in dispersal information is adequately described and acknowledged, making the decision process more transparent.

If time permits, and there is sufficient value in improving knowledge, a range of methods (Table S1) can reduce uncertainty in movement knowledge and increase the likelihood of meeting management objectives (shift 3, Figure 1B). Quantitative allometric comparisons (i.e., dispersal–body size relationships, Schloss et al., 2012) could reduce uncertainty in movement knowledge by revealing the influence of dispersal on species’ responses to management. Some methods involve intensive collection and analysis of new movement data including tracking or mark–recapture to quantify movement distances and behavior, landscape analysis of gene flow and mechanistic or simulation modeling (e.g., Smith et al., 2016; Spiegel & Nathan, 2007). Such studies might take months or years to complete but can feed back into the decision‐making process to enable adaptive management (Figure 2).

## 4 ILLUSTRATING THE FRAMEWORK WITH A REAL‐WORLD CASE STUDY

We chose a major agri‐environment scheme to illustrate our framework because it is relevant to many ecosystems globally and movement knowledge is highly relevant but often not explicitly considered. The Australian Government's *Multiple Ecological Communities Stewardship Project* used competitive tenders to fund biodiversity conservation projects led by private land managers (Burns, Zammit, Attwood, & Lindenmayer, 2016). The project's objective was to improve the extent, connectivity, function and resilience of threatened ecological communities. The need to conserve features that increased structural connectivity across the landscape, such as paddock trees and vegetation remnants, was specifically acknowledged in the project design. To estimate the conservation value of potential sites and the likelihood of meeting the objective, a *Conservation Value Metric* was developed (Whitten, Doerr, Doerr, Langston, & Wood, 2010). The Metric was used, in conjunction with the bid, to estimate the relative return on investment for managing a site for 15 years. It was derived from information on the starting condition, threats, and opportunities to enhance site condition. It explicitly incorporated connectivity by including isolation as a threat, and management of buffers around patches and connecting landscape features as opportunities. Less isolated patches, or opportunities to improve connectivity through management, were consequently ranked with higher conservation value (Whitten et al., 2010).

Assumptions relating to the minimum required buffer widths and the effectiveness of management options were based on dispersal trait information in the literature and movement abilities of surrogate species (Burns et al., 2016). Surrogate species were predominantly woodland birds and mammals (Doerr, Doerr, & Davies, 2011) meaning the movement of most taxa was not incorporated (due to a lack of published information on other species). Furthermore, to allow many sites to be assessed across a broad spatial scale, connectivity was estimated using satellite imagery of vegetation structure in woodlands, which might have limited relevance to the actual movement of several species. Focusing on the project design, we explore how uncertainty in movement knowledge, and relevance of movement, could be managed to better achieve the Stewardship Project's objective of conserving threatened ecological communities.

### 4.1 Assessment and strategy selection

Using Figure 1 as a guide, we begin by determining our position in knowledge‐relevance space (the starting point for managing uncertainty in movement knowledge). Since basic information on species’ movements was limited (Burns et al., 2016) there is, by definition, low movement knowledge. This lack of knowledge could bias the Project's outcome because we do not know if woodland connectivity is a reliable representation of species’ movements. The Stewardship Project would therefore be on the “low” side of the *x*‐axis (Figure 1A). Where then do we lie on the *y*‐axis? How relevant are species’ movements to the design of the project? Improving ecological function, such as plant pollinator interactions, requires movement of pollinators across landscapes (Greenleaf, Williams, Winfree, & Kremen, 2007). Protecting endangered species requires specific habitat configurations to ensure individuals can move across the landscape (Baguette, Blanchet, Legrand, Stevens, & Turlure, 2013). Thus, movement knowledge might be highly relevant to the Stewardship Project objectives but we are uncertain of *how* relevant because knowledge is limited. This places us in the top left corner: we are uncertain if movement is relevant, and we have little knowledge about movement (strategy option iii, Figures 1 and 2). From here, our framework provides three management pathways: (1) manage the relevance of movement knowledge through sensitivity analysis, or manage the lack of knowledge by either (2) increasing robustness (*accept*) or (3) improving movement knowledge (*improve*).

Data on dispersal distances for indicator taxa and traits for a broad range of species in the system (Table S1) could be used to define plausible upper and lower bounds of movement values for use in sensitivity analysis. Conservation planning tools such as Marxan can account for spatially explicit movement for multiple species to define species and/or community connectivity (Magris, Treml, Pressey, & Weeks, 2016). The influence of a plausible range of community connectivity values on the Metric for different sites could therefore be evaluated. If alterations in movement did not change the scores, the current solution can be seen as insensitive to uncertainty in movement information, and our initial position in uncertainty space would shift to the bottom‐left of Figure 1A, and project managers could proceed directly to *Implementation* (Figure 2). If scores were highly sensitive to movement variation, we would remain in the top‐left of Figure 1A and the lack of knowledge should be managed. In the Stewardship Project, connectivity was used in calculating the conservation value of sites and, depending on its strength relative to other parameters, might influence funding allocations. Managing a lack of movement knowledge might therefore be worthwhile for project managers.

### 4.2 Managing lack of knowledge

Ideally, large‐scale investments like the Stewardship Project would have simultaneous research programs that use the funded sites to assess movement (Watson et al., 2017). For example, a genetic study could be completed for A$50–200K including technician labor for multiple species to investigate relative movement capacity (Puckett, 2017), while satellite tracking is becoming increasingly feasible and automated. Drawing together a range of movement research methods in conservation programs provides an opportunity to learn about the importance of movement to environmental decisions, which can feed back into adaptive management. Value‐of‐information analysis could determine if resolving uncertainties in movement knowledge (i.e., collecting and analyzing new data) would change decisions about how to allocate funds (Canessa et al., 2015). Optimization algorithms could be used to determine the gain in increase of connectivity for a range of budgets (i.e., to identify if uncertainty could be resolved with new information about multiple species’ movement, given budget constraints). A useful feature of such an analysis is that value can be expressed as the relevant performance metric, such as the Stewardship Project's Metric (Maxwell et al., 2015).

The Stewardship Project had a substantial budget but very tight timelines (12 months to design a A$70 m program, Burns et al., 2016), meaning that time constraints would most likely dictate whether to accept or improve movement knowledge. Under such time constraints, robustness analysis could identify management strategies with enough flexibility to perform well with severe uncertainty around movement knowledge. Info‐gap analysis for the Stewardship Project would combine a performance model (in this case, the Metric) with an uncertainty model which includes an unbounded uncertainty parameter around a best guess (Regan et al., 2005). Modeling severe uncertainty in decision‐making can allow managers to make decisions, which are immune to uncertainty and these might be different than decisions based on a model, which ignored uncertainty (Regan et al., 2005). Thus, the outcome of the Stewardship Project might take a different course if the performance measure was combined with an uncertainty model, potentially leading to better outcomes for biodiversity. Robustness analysis could be integrated into existing performance measures for future agrienvironment schemes where resources were available to complete full quantitative analyses. This would provide new knowledge about the risk involved in a decision, that is, how wrong a movement estimate can be before it affects the conservation outcome.

## 5 CONCLUSIONS

Rather than providing strict prescriptions, our framework provides a set of flexible options for ascertaining the relevance of movement knowledge and managing uncertainty when making environmental decisions. Characterizing and reducing uncertainty makes project designs more transparent and facilitates communication between decision‐makers (Kujala et al., 2013). Managing uncertainty is a key part of structured decision‐making (Gregory et al., 2012) and our framework could be used in this part of the decision process to improve the biodiversity outcomes of systematic conservation planning, reserve design, agri‐environment schemes, habitat restoration, and assisted migration. The importance of movement knowledge might be contingent on other aspects of the system or decision‐making processes, which may also be uncertain (Kujala, Whitehead, Morris, & Wintle, 2015). Frameworks for dealing with uncertainty in future climate and sociopolitical environments could be drawn on to inform complex, multifaceted environmental decisions (Schwartz et al., 2018). Finally, improving fundamental knowledge on movement in plants and animals will enhance the capacity for policy and management decisions to conserve natural movement processes in future (Driscoll et al., 2014).

## Supporting information


**Table S1**. Methods for using and improving movement knowledge for environmental decisions. The list is nonexhaustive and focuses on methods relevant to our framework and case study.
