# Supplementary material for: Managing uncertainty in movement knowledge for environmental decisions
Source: Conserv Lett. 2018 Dec 3;12(3):e12620. doi: 10.1111/conl.12620 (PMC6686712; doi:10.1111/conl.12620)
Supplement: Supplementary file 1 — Table S1. Methods for using and improving movement knowledge for environmental decisions. The list is nonexhaustive and focuses on methods relevant to our framework and case study. [file CONL-12-na-s001.docx]

**Managing uncertainty in movement knowledge for environmental decisions**

Annabel L. Smith^1,*^, Heini Kujala^2^, José J. Lahoz-Monfort^2^, Lydia K. Guja^3,4^, Emma L. Burns^5,6^, Ran Nathan^7^, Erika Alacs^8^, Philip S. Barton^5^, Sana Bau^2^, Don A. Driscoll^9^, Pia E. Lentini^2^, Alessio Mortelliti^10^, Ross Rowe^11^ and Yvonne M. Buckley^1,12^

^1^ School of Natural Sciences, Zoology, Trinity College Dublin, The University of Dublin, Dublin 2, Ireland

^2^ School of BioSciences, The University of Melbourne, Melbourne 3010, Australia

^3^ Parks Australia Division, Department of the Environment and Energy, Canberra 2600, Australia

^4^ Centre for Australian National Biodiversity Research, CSIRO, Canberra 2601, Australia

^5^ Fenner School of Environment and Society, Australian National University, Canberra 2601, Australia

^6^ Long Term Ecological Research Network, Terrestrial Ecosystem Research Network, Australia.

^7^ Department of Ecology, Evolution and Behavior, The Alexander Silberman Institute of Life Sciences, The Hebrew University of Jerusalem, Jerusalem 91904, Israel

^8^ Wildlife Heritage & Marine Division, Department of the Environment and Energy, Canberra 2600, Australia

^9^ School of Life and Environmental Sciences, Deakin University Geelong, Burwood 3125, Australia

^10^ Department of Wildlife, Fisheries, and Conservation Biology, University of Maine, Orono 04469 USA

^11^ Environment Standards Division, Department of the Environment and Energy, Canberra 2600, Australia

^12^ School of Biological Sciences, University of Queensland, St Lucia 4072, Australia

* Correspondence: annabel@smithecology.org

**Table S1.** Methods for using and improving movement knowledge for environmental decisions. The list is non-exhaustive and focusses on methods relevant to our framework and case study.

| **#** | **Method** | **Key assumptions relevant to environmental decision making** | **Estimated variable** | **Pros** | **Cons** | **Example references** |
| --- | --- | --- | --- | --- | --- | --- |
| 1 | Vegetation connectivity maps | Vegetation connectivity is positively related to species’ movement; structural connectivity might reflect functional connectivity | Connectivity / isolation; landscape permeability / resistance | Easy to collect via satellite imagery; might reflect movements of several species | Might not reflect actual movements; difficult to obtain for ecosystems that do not have strong structural differences to the matrix (e.g. grasslands) | (Doerr et al. 2011; Muratet et al. 2013) |
| 2 | Surrogate / indicator species | Dispersal of one species will reflect that of another | Occupancy / trait congruence | Does not require collection of new data | Key assumption (i.e. that a given species is a valid proxy) is not well established | (Lentini and Wintle 2015; Pierson et al. 2016) |
| 3 | Dispersal traits / allometry | Body mass, morphology, propagule size (e.g. seed mass and in plants, leg / wing length animals) can predict movement distances and dispersal syndrome | Mean / max displacement distance; Relative dispersal distance; dispersal syndrome | Data can be collected from online databases (e.g. Kew Seed Information Database); easy to implement if allometric relationships are known for relevant functional group; movement can be estimated with no further measurements | Very coarse, typically wide variance around the estimated variable; relevant allometric relationships might not be available; mainly useful in a comparative sense | (Jenkins et al. 2007; Moles et al. 2005; Schloss et al. 2012) |
| 4 | Telemetry and mark-recapture | Recorded movements will reflect functional effects, such as movement from birth place to place of reproduction | Home-range size; mean / max dispersal distance | Directly measures movement | Difficult to measure; usually constrained to large organisms / propagules; long-distance dispersal events often missed | (Cooke et al. 2004; Hassall and Thompson 2012; Suselbeek et al. 2013) |
| 5 | Genetics and genomics | Spatial genetic structure arises from neutral gene flow and reflects actual movements and matings of individuals rather than environmental adaptation | Genetic distance between individuals, populations or species; neighbourhood size | Does not require observation or tracking of individuals; can incorporate movements over successive generations (which can also be a con); can give insights into other biological processes such as mating system and parentage | Can reflect historical rather than contemporary gene flow; data can be expensive and time consuming to produce; signals from adaptation and demographic variation could be confused with effects of movement | (Moran and Clark 2012; Steinitz et al. 2012) |
| 6 | Phenomenological movement kernels | Movement *patterns* in the entire system are similar to the movement quantified in part of the system or elsewhere; movement is isotropic and probability of displacement depends only on Euclidean distance between source and end points | Displacement kernel | Easy to implement; more robust to model uncertainty than mechanistic movement kernels | May require additional measurements; extrapolation beyond the (typically spatially limited) observed data not well justified; parameter uncertainty might be relatively high | (Clark et al. 2001; Kremer et al. 2012) |
| 7 | Mechanistic movement kernels | Movement *mechanisms* in the entire system are similar to the movement quantified in part of the system or elsewhere; the probability of displacement depends only on Euclidean distance between source and end points. | Displacement kernel | Relatively general for each major movement process being modeled; can be adjusted in a relatively simple manner to the specific features of the focal system (e.g. anisotropy) | May require additional measurements or complicated modeling; model uncertainty might be relatively high (movement might be driven by processes other than the one chosen for the mechanistic model) | (Damschen et al. 2014; Spiegel and Nathan 2007) |

**References**

Clark, J.S., Lewis, M., Horvath, L. (2001). Invasion by extremes: population spread with variation in dispersal and reproduction. The American Naturalist **157**, 537-554.

Cooke, S.J., Hinch, S.G., Wikelski, M., Andrews, R.D., Kuchel, L.J., Wolcott, T.G., Butler, P.J. (2004). Biotelemetry: a mechanistic approach to ecology. Trends in Ecology & Evolution **19**, 334-343.

Damschen, E.I., Baker, D.V., Bohrer, G., Nathan, R., Orrock, J.L., Turner, J.R., Brudvig, L.A., Haddad, N.M., Levey, D.J., Tewksbury, J.J. (2014). How fragmentation and corridors affect wind dynamics and seed dispersal in open habitats. Proceedings of the National Academy of Sciences **111**, 3484-3489.

Doerr, V.A.J., Doerr, E.D., Davies, M.J. (2011). Dispersal behaviour of brown treecreepers predicts functional connectivity for several other woodland birds. Emu **111**, 71-83.

Hassall, C., Thompson, D.J. (2012). Study design and mark-recapture estimates of dispersal: a case study with the endangered damselfly *Coenagrion mercuriale*. Journal of Insect Conservation **16**, 111-120.

Jenkins, D.G., Brescacin, C.R., Duxbury, C.V., Elliott, J.A., Evans, J.A., Grablow, K.R., Hillegass, M., Lyon, B.N., Metzger, G.A., Olandese, M.L., Pepe, D., Silvers, G.A., Suresch, H.N., Thompson, T.N., Trexler, C.M., Williams, G.E., Williams, N.C., Williams, S.E. (2007). Does size matter for dispersal distance? Global Ecology and Biogeography **16**, 415-425.

Kremer, A., Ronce, O., Robledo-Arnuncio, J.J., Guillaume, F., Bohrer, G., Nathan, R., Bridle, J.R., Gomulkiewicz, R., Klein, E.K., Ritland, K., Kuparinen, A., Gerber, S., Schueler, S. (2012). Long-distance gene flow and adaptation of forest trees to rapid climate change. Ecology Letters **15**, 378-392.

Lentini, P.E., Wintle, B.A. (2015). Spatial conservation priorities are highly sensitive to choice of biodiversity surrogates and species distribution model type. Ecography **38**, 1101-1111.

Moles, A.T., Ackerly, D.D., Webb, C.O., Tweddle, J.C., Dickie, J.B., Pitman, A.J., Westoby, M. (2005). Factors that shape seed mass evolution. Proceedings of the National Academy of Sciences of the United States of America **102**, 10540-10544.

Moran, E.V., Clark, J.S. (2012). Between-site differences in the scale of dispersal and gene flow in red oak. PLoS ONE **7**, e36492.

Muratet, A., Lorrillière, R., Clergeau, P., Fontaine, C. (2013). Evaluation of landscape connectivity at community level using satellite-derived NDVI. Landscape Ecology **28**, 95-105.

Pierson, J.C., Mortelliti, A., Barton, P.S., Lane, P.W., Lindenmayer, D.B. (2016). Evaluating the effectiveness of overstory cover as a surrogate for bird community diversity and population trends. Ecological Indicators **61**, 790-798.

Schloss, C.A., Nuñez, T.A., Lawler, J.J. (2012). Dispersal will limit ability of mammals to track climate change in the Western Hemisphere. Proceedings of the National Academy of Sciences **109**, 8606-8611.

Spiegel, O., Nathan, R. (2007). Incorporating dispersal distance into the disperser effectiveness framework: frugivorous birds provide complementary dispersal to plants in a patchy environment. Ecology Letters **10**, 718-728.

Steinitz, O., Robledo-Arnuncio, J.J., Nathan, R. (2012). Effects of forest plantations on the genetic composition of conspecific native Aleppo pine populations. Molecular Ecology **21**, 300-313.

Suselbeek, L., Jansen, P.A., Prins, H.H.T., Steele, M.A. (2013). Tracking rodent-dispersed large seeds with Passive Integrated Transponder (PIT) tags. Methods in Ecology and Evolution **4**, 513-519.
